# HEARING LOSS AND COGNITIVE DECLINE: EVIDENCE FROM THE LONGITUDINAL STUDY OF ADULT HEALTH (ELSA-BRASIL)

**DOI:** 10.1093/geroni/igae098.0314

**Published:** 2024-12-31

**Authors:** Natalia Gomes Goncalves, Alessandra Samelli, Paulo Lotufo, Isabela Bensenor, Claudia Suemoto

**Affiliations:** University of São Paulo Medical School, São Paulo, Sao Paulo, Brazil; University of São Paulo, São Paulo, Sao Paulo, Brazil; University of São Paulo, São Paulo, Sao Paulo, Brazil; University of São Paulo, São Paulo, Sao Paulo, Brazil; University of São Paulo, São Paulo, Sao Paulo, Brazil

## Abstract

Hearing loss is common in older adults and its prevalence increases with age, being present in 40% of adults aged 50 years or older and increasing to 70% in adults aged 70 years or older. Hearing loss in middle age is one of the main risk factors for dementia, and it is estimated that 7% of dementia cases in Brazil are due to hearing loss. Therefore, this study aimed to study the association between hearing loss and cognitive decline during 8 years of follow-up. Data included participants from the São Paulo center of the Longitudinal Study of Adult Health (ELSA-Brasil), who were evaluated in three study waves (2008-10, 2012-14, and 2017-19). Hearing loss was defined as pure-tone audiometry above 25 dB in either ear. Six tests evaluated cognitive performance in the memory, verbal fluency, and executive function domains. A global cognitive z-score was derived from these tests. The association between hearing loss and cognitive decline was evaluated with linear mixed-effects models adjusted for sociodemographic, lifestyle, and clinical variables. Of 805 participants (mean age 50.6±9.4 years, 52% women, 60% White), 62 had hearing loss. During follow-up, hearing loss was associated with faster global cognitive decline (β=-0.012, 95% CI=-0.023; 0.000, p=0.039). These results suggest that prevention of hearing loss in middle age could contribute to slowing cognitive decline in this population.

